# Training and development experiences of nursing associate trainees based in primary care across England: a qualitative study

**DOI:** 10.1017/S1463423623000221

**Published:** 2023-04-28

**Authors:** Rachel King, Sara Laker, Sarah Alden, Tony Ryan, Emily Wood, Angela Tod, Michaela Senek, Bethany Taylor, Steven Robertson

**Affiliations:** 1 RCN Strategic Research Alliance, Division of Nursing & Midwifery, Health Sciences School, Barber House Annexe, 3a Clarkehouse Road, Sheffield S10 2LA, UK; 2 Department of Nursing, Winona State University, Stark Hall, Room 303, Winona, MN 55987, USA; 3 Leeds Beckett University, Leeds, UK; 4 Waterford Institute of Technology, Waterford, Ireland

**Keywords:** education nursing, health workforce, nursing associates, primary health care, United Kingdom

## Abstract

**Background:**

The nursing associate role was first deployed in England in 2019 to fill a perceived skills gap in the nursing workforce between healthcare assistants and registered nurses and to offer an alternative route into registered nursing. Initially, trainee nursing associates were predominantly based in hospital settings; however, more recently, there has been an increase in trainees based in primary care settings. Early research has focussed on experiences of the role across a range of settings, particularly secondary care; therefore, little is known about the experiences and unique support needs of trainees based in primary care.

**Aim:**

To explore the experiences and career development opportunities for trainee nursing associates based in primary care.

**Methods:**

This study used a qualitative exploratory design. Semi-structured interviews were undertaken with 11 trainee nursing associates based in primary care from across England. Data were collected between October and November 2021, transcribed and analysed thematically.

**Findings:**

Four key themes relating to primary care trainee experiences of training and development were identified. Firstly, nursing associate training provided a ‘valuable opportunity for career progression’. Trainees were frustrated by the ‘emphasis on secondary care’ in both academic content and placement portfolio requirements. They also experienced ‘inconsistency in support’ from their managers and assessors and noted a number of ‘constraints to their learning opportunities’, including the opportunity to progress to become registered nurses.

**Conclusion:**

This study raises important issues for trainee nursing associates, which may influence the recruitment and retention of the nursing associate workforce in primary care. Educators should consider adjustments to how the curriculum is delivered, including primary care skills and relevant assessments. Employers need to recognise the resource requirements for the programme, in relation to time and support, to avoid undue stress for trainees. Protected learning time should enable trainees to meet the required proficiencies.

## Introduction

In 2019, the first nursing associates (NAs) not only entered the healthcare workforce in England, principally as a bridging role between the healthcare assistant (HCA) and registered nurse (RN) role but also to provide an alternative route into registered nursing (HEE, 2015). Initially, trainees were predominantly based in larger hospital trusts in medical and surgical wards (Vanson and Bidey, 2019, Kessler *et al*., 2020); however, more recently, there has been an increase in those based in private, independent and voluntary sector organisations, including GP practices. This has been enabled through recent incentives provided to employers through apprenticeship funding (ESFA, 2021). Previous research has identified unique challenges for these trainees from the perspective of university staff involved in delivering the programmes (Robertson *et al.*, 2022; Robertson *et al.,* 2023). Others have described the difficulties in developing an appropriate curriculum for NA training (Roulston and Davies, 2019). However, little is known about the learning, support and career development experiences of trainees in those settings. This paper presents key findings from interviews with trainee NAs based in primary care from across England. As Robertson *et al*. (2023) note, in the UK, the term primary care is commonly used to signify those services delivered by general practices (GPs), and this is the way the term was understood in this study and in this paper.

## Background

The NA role is becoming well-established in England, with initial uncertainty about the role among employers reducing (Kessler *et al.,* 2021). Similar second-level nursing roles are embedded in healthcare teams in several other high-income countries, such as enrolled nurses in Australia and New Zealand and licenced practical nurses (LPN) in North America. This is not the first time there has been a second-level nursing role in England; the new NA role has been compared by some to the previously enrolled nurse role in the UK (Lucas *et al.*, 2021a; The Health Foundation Nuffield Trust, The King’s Fund, 2018).

Following a 2-year diploma-level programme, second-level nurses generally work under the supervision of registered nurses providing fundamental nursing care (Lucas *et al.,* 2021a). The NA programme includes a base placement with the employer and shorter alternative placements across the four fields of nursing (adult, child, mental health and learning disability) and a range of settings (NMC, 2018a). However, the scope of NA practice in England varies widely, with many administering medication via oral, subcutaneous and intramuscular routes, providing complex wound care and catheterising patients (Kessler *et al.,* 2021). In contrast to other similar-level healthcare roles which are also paid at ‘Agenda for Change’ band 4 (NHS Employers, 2020), such as assistant practitioners (Kessler and Nath, 2019), NAs are registered with the Nursing and Midwifery Council (NMC, 2018b).

There are opportunities, via the apprenticeship route, to build on the NA training to become RNs; a route supported by Health Education England’s vision for progression in the primary care nursing workforce (HEE, 2017). In their focus group study, King *et al*. (2020) suggest a key motivator for many entering training was this opportunity for career progression. Furthermore, a national evaluation of the NA pilot programmes, found that 70% of trainee NAs expressed a desire to become registered nurses (Vanson and Bidey, 2019). Importantly though, subsequent research highlights that these career progression opportunities are often influenced by local context, with most newly qualified NAs undertaking a preceptorship programme rather than moving directly into registered nurse training (Kessler *et al.*, 2020a). Some organisations that are keen to grow their own workforce in managing workforce shortages require the NAs to stay working in the role for a period of time after qualification with some mandating this (Kessler *et al.*, 2020b, 2020c).

King *et al.* (2022) identified key factors important to trainee and early career NAs in their decision-making about career progression. Some who chose to pursue RN training were driven by their experiences of role ambiguity and interprofessional conflict, while others showed a clear desire and plan for career progression. For those who chose to remain as NAs, some had faced barriers to accessing further training, and others felt that their age prevented them from embarking on further training, whilst some preferred to embed the new NA role.

There is growing evidence of the challenges in embedding new roles in healthcare, particularly where professional boundaries become blurred and the scope of practice varies across settings (Nancarrow and Borthwick, 2005; King *et al.,* 2021). In the case of second-level nurses, such challenges include role ambiguity, intra and inter-professional conflict and restrictions on practice and development (Kessler *et al.,* 2021; Lucas *et al.,* 2021a; 2021b). This may explain the desire by many trainees and qualified NAs (73%) to transition into registered nursing, although this progression is not always supported by employers who are often keen to embed the NA role in organisations (Kessler *et al.,* 2021). Other barriers to career progression have been identified in those accessing modern apprenticeships, such as finance, employer support and perceptions of their own abilities (Bowers-Brown and Berry, 2005). Furthermore, career development opportunities were dependent on employer knowledge and agendas, with fewer options to progress amongst the smallest employers.

Previous research has explored the experiences of trainee (Coghill, 2018a, 2018b; Vanson and Bidey, 2019; King *et al*., 2020, 2021), and newly qualified NAs (Kessler *et al.,* 2020, 2021; Lucas *et al.,* 2021b), yet there were few participants from primary care in these early studies, and their experiences have therefore yet to be reported. The number of such trainee NAs have been increasing since changes to apprenticeship funding rules in October 2020 (ESFA, 2020; Robertson *et al.*, 2022; Robertson *et al.*, 2023). This qualitative study builds on previous research by specifically exploring the motivations, learning experiences, and career aspirations of trainee NAs based in GP surgeries in primary care (GPs) in England.

## Methods

This study used a qualitative exploratory design. Three researchers undertook the interviews, one male and two female (RK, SR, and SA), all with experience in healthcare research, and two with registered nursing experience in both primary and secondary care. Reporting has followed the COREQ criteria (Tong *et al*., 2007).

Five higher education institutions (HEIs) situated in the North West (n = 1), Yorkshire (n = 2), Midlands (n = 1), and the South East (including London) (n = 1) were contacted to support recruitment. HEIs were purposively chosen to provide a diversity of geographical locations and approaches to NA training. Course leaders distributed information about the study to NA cohorts that were more than 6 months into their training. The information made it clear that the study related to trainees in primary care and volunteers provided written consent. The email contained a link to the information sheet and consent form. Trainees were asked to provide a contact email address on the consent form and to indicate their region to facilitate purposive sampling. Participants were contacted by email to arrange a convenient time for the interview. Non-responders were contacted twice (after 1 week, then after 1 more week). Each participant was offered a £10 shopping voucher as a thank you for their time.

Eleven interviews were undertaken between October and November 2021 via video call (Google Meet) using a topic guide (see Box 1). Interviews lasted between 25 and 51 min and were audio recorded and transcribed using professional transcribers. Trainee NA (TNA) participants were based in the Northwest (n = 3), West Yorkshire (n = 1), South Yorkshire (n = 5), and the South East (n = 2). All 11 participants were female, nine identified as white British, with one white other and one British Asian. Three were in their 20’s, five in their 30’s, two in their 40’s, and one in their 50’s.

Box 1. Interview Topic guide

1. Demographic questions (gender, age, ethnicity)
2. Motivations for commencing training
3. Training experiences (academic and clinical training, protected learning time, support, intention to leave)
4. Workplace identity (own, response of patients and colleagues to the role)
5. Career aspirations (short term and long-term plans)

In addressing the issue of data saturation, the concept of information power was applied to the sample size of eleven (Malterud *et al*., 2016). This takes account of the study aim, sample specificity, application of established theory, quality of dialogue, and method of analysis. The research team already had some insight into the training experiences of NAs in a range of health and social care settings, and associated theory, from their previous research (anon) which helped to inform the development of the interview guide. Recruitment had a narrow focus on trainees based in primary care, therefore workforce characteristics were very specific to the aims of the study.

Data analysis by two experienced researchers (RK and SR) followed the six-step process of thematic analysis outlined by Braun and Clarke (2006); familiarisation with the data, generating initial codes, searching for themes, reviewing themes, defining and naming themes, and producing the report. Quirkos© computer-assisted qualitative data analysis software (CAQDAS) was used to help manage the data analysis. Data were anonymised prior to uploading to Quirkos©. Ethical approval was granted from the University research ethics committee (Ref: 043079).

## Findings

This paper presents the key issues important to trainee NAs based in primary care, general practice, settings in England (see Box 2 for themes and sub-themes).

Box 2. Themes and sub-themes

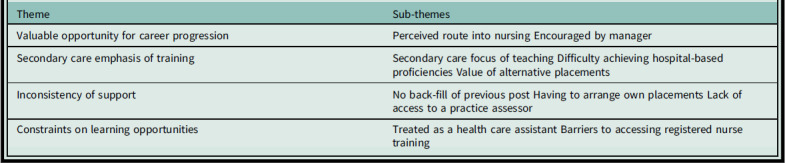

### Theme 1: valuable opportunity for career progression

All 11 participants noted the valuable opportunity for career progression afforded by the programme; a natural progression for those with experience as HCAs. They described how NA training had provided an opportunity to gain knowledge and skills beyond that offered in their previous roles. It was also viewed by most participants as an affordable route into registered nursing:

*‘I’ve always wanted to do nursing, it’s been like my main goal since I was little. And then various things have happened where I’ve not actually managed to do it, and I’ve just sort of stayed in a HCA role for longer than I really wanted to. And then this just felt like an easier step… I’ve got a family, I can’t afford to go away and be a student and not earn any money.’ TNA 2*

*‘I’d always wanted to do my nurse training, but I couldn’t afford to leave work completely, and especially after taking the bursary away, you know, I couldn’t afford to have debts at the end of it so it was a good first step for me, potentially, doing my full nurse training.’ TNA 7*

In the majority of cases, after initial fact-finding work by the potential trainee, employers actively encouraged their staff to apply to the programme. For some, though it was a more difficult process. One trainee described how she had to persuade her employer that it was a worthwhile option, particularly in terms of it being cost-effective for the practice organisation in the long run. However, there is perhaps a misrepresentation of the role here; as a substitute for practice nursing:

*‘I think the GP practice that I’m based at, the GP there is quite involved with the PCN [primary care network] … he’s got quite an interest in the NA roles and he’s the one who actually told me about the role. He knew quite a bit about the role and encouraged me to apply for it. He goes out of his way.’ TNA 5*

*‘I sourced the funding directly with Health Education England, presented it to my employer who was quite keen to get me on the training as well.’ TNA 3*

*‘I did a bit of research myself, and kind of went out and found different job specs from different Nursing Associates in different parts of the country, so me and my Nurse Practitioner did that. So, I went to them and said well this is why you should let me do the course, because when I qualify I’ll be able to do x, y, z, you know? So you potentially wouldn’t have to employ another practice nurse, you know, ‘cause we can manage the workload.’ TNA 7*

Generally, after this initial fact-finding, the NA training programme was viewed as an exciting opportunity by both trainees and their primary care employers. However, there were particular hurdles for trainees based in primary care, which they expressed by comparing their experiences to the majority of their peers who were based in larger secondary care settings.

### Theme 2: secondary care emphasis of NA training

Several participants talked about the lack of relevance of NA training to the primary care setting, describing a greater emphasis on secondary care in both the academic content, the competencies required, and the methods of assessment in placement. Some felt this was due to a general lack of understanding of the NA role in primary care. One strategy to overcome their knowledge gaps during the academic study time was to seek clarification from their secondary care-based peers on the programme:

*‘When they are doing the lectures, and when they are giving you scenarios, it’s always a hospital scenario and they use different nursing tools to us. It’s always abbreviated, and you are like, what does this mean… luckily I know somebody on the course who works at the hospital, so I’m like, “What’s this?”’ TNA 1*

*‘A lot of the learning outcomes, proficiencies and things like that are just more achievable in a secondary care setting. And we’re never given any examples on how they want us to achieve it, it’s always given as if we’re from secondary care. And it’s like, “no, we’re still here, we’re from primary care, we can’t do it that way, could you tell us how to do it this way.”’ TNA 3*

*‘I find university doesn’t match up at all with what I need to learn and I find that the lecturers at university don’t really know… they call it GP-land…they don’t really know what we should be doing, what’s relevant to us. So I find the university side quite frustrating.’ TNA 9*

As a consequence of the secondary care focus, it was difficult for trainees based in primary care to meet some of the requirements of the programme while on placement. For example, one participant described a lack of opportunities to gain experience in drug administration to meet the medicines management competency and another talked about challenges in reflecting on certain aspects of care, irrelevant to primary care, such as patient discharge:

*‘People in hospitals probably do medicine management much easier because they’re dealing with medicine. But in primary care we don’t dispense medicine within the practice, it was a bit of a tricky one.’ TNA 5*

*‘I’m trying to do a workbook, and they are saying to relate it to your own workplace. But one of them is discharge. We don’t do discharge in a GP surgery. So, I’m having to write that, obviously, we don’t do it, but if I was in a hospital base, this is what I would do.’ TNA 8*

They relied on their alternative placements to meet many of the proficiencies which were not attainable in their primary care setting. This often required a tenacious attitude in seeking opportunities to experience a variety of skills in a short period of time:

*‘You really need to be looking at like going out there and actively trying to achieve these proficiencies, these professional values, because you’re not going to see it all in your base setting. So you really need to advocate for yourself to get the best of this experience.’ TNA 10*

Despite the challenges described in relation to the secondary care emphasis of training, some participants spoke of the value of their alternative placements. They demonstrated how these alternative placements helped increase their understanding of issues faced in primary care and also broadened their awareness of potential career opportunities:

*‘You know, obviously, a lot of procedures that they do in hospitals we wouldn’t do within a surgery. Which is good to know what they are, so at least when you see patients you can give them information and tell them, you know, “This is what’s going to happen.” Whilst, if I’ve never seen it, I can’t give that information.’ TNA 8*

*‘That was gynaecology outpatients [placement], and that was really interesting and relevant to my base placement, just to learn more about gynaecology and, obviously, hopefully when I complete the course, I’ll be doing smears.’ TNA 11*

*‘I’ve only ever come from a GP background, I’ve never had that acute setting or that inpatient experience. I’ve gone onto wards, I’ve gone to A and E, and I’ve just found different aspects that I enjoy. It really opened my eyes and gave me a lot of understanding as to the other roles out there that I could possibly see myself doing in the future.’ TNA 10*

Clearly, these primary care trainees felt there was an over emphasis on secondary care in their programmes; however, despite this, some enjoyed the breadth of clinical experiences that they were exposed to. Although valuable learning took place during the alternative placements, trainees often faced difficulties in accessing the required support while in their primary care employment base.

#### Theme 3: Inconsistency of support

Despite initial encouragement to undertake the training, some participants felt that their primary care managers did not fully appreciate the time commitment or supervision required of the programme. One talked about a lack of funding for backfill of her post, and a subsequent feeling of distress, knowing that patient care was adversely impacted when she was away from her base and on alternative placements. Another had not been given any time in her contracted hours to attend university and alternative placements:

*‘What I’m finding the most difficult is the whole aspect of when I’m out on placement, I’m not here and there is no backfill for my role, there’s nobody else that picks up my responsibilities or is here to see my patients when I’m not here and they need to be seen by somebody.’ TNA 3*

*‘The practice was supportive at the beginning but once they found out about how much time I needed to be on placements and release for some study time during the week, then there was the misunderstanding with the money, what to expect, you know? I’m doing two days of university. On top of that, I’m doing 30 hours of work. Last year, after failing the exam, I spoke to the two partners at the surgery and I said, “I think I’m about to quit altogether.”’ TNA 4*

In addition to misunderstandings about placement and study hours, there was also inconsistency in access to supervision. Some participants had positive experiences of supervision, although most noted difficulties in finding opportunities to work alongside supervisors and assessors in their base placements. In some cases, they had to request support from practice assessors from another organisation:

*‘My hub, where I am based, they are my blanket, my comfort blanket. They have been so supportive from the practice manager, the GPs, the nurses, the whole team really have been really supportive.’ TNA 1*

*‘My only problem with my assessor this year is that she is now doing a course herself, so her time is limited. So, I’ve now got to speak to the practice manager and say, “is it okay for me to use the other practice assessor from the other surgery?”’ TNA 8*

*‘The problem is, I don’t essentially have a PA [practice assessor], so I don’t have a person that’s assigned to me, as an education facilitator, because I don’t work for a trust. So, I think that needs to be evened out, ‘cause I mean… not that I need to talk to someone very often, but sometimes it’s useful just to bounce a few ideas off somebody that deals with the role day in day out you know?’ TNA 7*

This inconsistency of support was also evident in the constraints on learning and development opportunities experienced by several of the TNA participants during their training.

### Theme 4: constraints on learning and development opportunities

When working in their base placements, many trainees faced the tension of continuing their role as HCA while simultaneously being a learner. Some felt a responsibility to provide the usual care to patients, as they had done in their previous HCA roles. This gave them minimal opportunities to learn from nursing colleagues due to the isolated nature of primary care work:

*‘If I want to spend time with the nurse, I’ll have to come in on my day off to do that. So, because they need me for those two days. You are meant to get 20 percent time with your assessor, or your nurse, to help you; but, I can understand why the surgery can’t give me that, because, if I took 20 percent out of my 15 hours that I’m there I’m not going to get anything done!’ TNA 8*

Unfortunately, several participants were also expected to work as HCAs during their alternative placements, particularly in times of short staffing during peaks in the COVID-19 pandemic. Some also felt overlooked by supervisors who appeared to prioritise undergraduate degree-level student nurses when learning opportunities arose. This further impacted their ability to achieve secondary care-focussed proficiencies:

*‘There were times where I was just told to go special; sit outside someone’s room and make sure they don’t fall over for three hours… I did keep going back and saying, “is there anything you’d like me to do, do you want me to do the obs [observations], do you want me to follow a nurse around?” But for them, it was an extra pair of hands, to be honest with you, at the best of times.’ TNA 5*

*’When I was on lower GI surgery they just used me as a healthcare, like a support worker really. Which I’m happy to do, I’m there to learn I’m not turning my nose up at anything but it varies how much learning there is to be had, depending on where you are.’ TNA 9*

*‘So when it comes to like going on placement, if there’s student nurses, they’ll be doing all the ward rounds and I’ll be doing the beds and stuff like that.’ TNA 10*

There were further constraints on learning and development in relation to accessing registered nurse training; an aspiration of most of the participants. Several described their uncertainty about whether they would be funded to do the extra training while working in primary care:

*‘Well, the original plan was never to stop at nursing associate, it was always to do the additional training for registered nurse, but I don’t think it will happen here, if I’m honest with you.’ TNA 4*

*‘There probably will be ways of getting round it and self-funding if I need to, but again, it’s all going to depend on figures, can I afford my rent, can I afford my car lease, can I afford the car that I’m going to need to get me to and from a placement and to and from university.’ TNA 3*

One was more optimistic about being able to ‘top up’, stating: *‘Work are pretty supportive, so I don’t think there’ll be a problem from that point of view.’ TNA 7*

These barriers not only impacted the overall learning and development experiences of trainee NAs based in primary care, but they also exacerbated the challenges related to achieving placement requirements outside of the larger secondary care trust settings.

## Discussion

As the nursing associate role is rolled out across a wide range of health and social care settings in England, it is crucial to understand the experiences of trainees in different contexts. This study has identified key issues important to trainee NAs based in primary care, which will inform future policy and practice with the aim to improve recruitment and retention at a time where healthcare workforce shortages, exacerbated by the covid-19 pandemic, are of great concern (King *et al.*, 2021). The small nature of GP-independent businesses means that opportunities for meeting learning requirements of the programme are difficult for trainee NAs based in primary care at times of high service need.

The trainees in this study are largely motivated by career progression and an affordable route into nursing, which supports the findings from previous studies (Lucas *et al.,* 2021b; King *et al.,* 2020). In addition to personal motivation, most also received encouragement to apply to the programme from their employers who have recently been offered financial incentives to take on new apprentices (ESFA, 2021). Consequently, an increasing number of trainees are based in primary care.

This study has identified a lack of understanding by some primary care managers of the time commitment for NA trainees to attend university and alternative placements during the apprenticeship, an issue which has also been identified by Kessler *et al*. (2020) in their research, largely focused on hospital settings. It is crucial that mangers are clear about the requirements of the programme as absences for attending lectures and alternative placements will potentially have a greater impact on service provision in primary care compared to secondary care, due to the smaller size of the workforce in those organisations.

One of the main frustrations about the training for these participants based in primary care was the focus on secondary care knowledge, skills, and assessments. They felt there was often a lack of recognition of their very different scope of practice and concomitant training needs. One reason for the focus on secondary care by universities might be that when NA training programmes were initially designed, the majority of trainees came from hospital settings (Vanson and Bidey, 2019; Kessler *et al.,* 2020). The proficiencies, aimed to assess NAs for a generic role across all four fields of nursing, have been set by the Nursing and Midwifery Council (NMC, 2018c); however, for participants in this study, the majority of training hours are undertaken in their primary care base placements. This creates a challenge for trainees from the start of the programme.

Concerns related to resources within base placements, such as a lack of backfill for HCA work, and a lack of support from practice assessors, were raised by some participants. One solution to the precarious nature of the availability of practice assessors in smaller surgeries, suggested by Robertson *et al.* (2022), is to share resources across the larger Primary Care Networks in areas where these exist. This is not only important in meeting the proficiencies, but also providing time to undertake other assessments, such as medicines management and observing episodes of care.

Opportunities for career development beyond the NA training were dependent on employer workforce planning and often provided further challenges for those keen to progress to become registered nurses. Lucas *et al.* (2021b) emphasise that the NA role should be seen as both a step into registered nursing and a role in itself. Support from the employer, in addition to support from HEIs, has been shown as crucial to apprentice career development (DfES, 2003). This is also consistent with research by Kessler *et al*. (2021), who found that opportunities for development offered by employers did often not always align with the desire of NAs to transition to RN. In small primary care settings, it is essential that employer workforce planning intentions and NA aspirations are clearly discussed at the earliest opportunity to avoid misunderstandings and disappointment.

## Limitations

This sample of 11 trainee nursing associates from primary care was geographically diverse and included a range of ages. Despite this geographical diversity, the qualitative nature of the study, including the small sample, does not make a comment on variation by region possible or meaningful. There was also a lack of diversity in terms of ethnicity and gender which may mean that some views and experiences are not considered here. The limited ethnic diversity in the sample is particularly disappointing given the potential for social mobility that NA training provides. One report indicates that a quarter of trainee nursing associates are not white (Vanson and Bidey, 2019). Future recruitment strategies for studies of trainee NAs could either focus specifically on under-represented groups or ensure fuller representation across geographical regions, genders, and ethnic groups.

## Conclusion

This study has revealed some important challenges for trainee NAs based in primary care which could help inform both workforce planning and academic support. It is crucial that the approach to the delivery of the curriculum includes primary care skills and relevant assessments. It is also important that GP employers are familiar with the resource requirements for the programme and seek collaboration across practices to optimise support for trainees. Practice supervisors and assessors should be made aware of the challenges presented in the findings, especially those related to difficulties in meeting programme proficiencies and a lack of resources to backfill posts. They must take account of the isolated nature of working in primary care and provide relevant learning opportunities in both base and alternative placements to ensure that required proficiencies can be met. In order to mitigate difficulties in meeting secondary care competencies, primary care-based trainee nursing associates require enhanced support in both their base and alternative placements.
